# Are physical activity, sleep, and joint pain associated with physical function and quality of life in individuals with multimorbidity? A cross-sectional analysis of the MOBILIZE trial

**DOI:** 10.1007/s11136-025-04044-4

**Published:** 2025-08-09

**Authors:** Travis Haber, Alessio Bricca, Michelle Hall, Jan Christian Brønd, Lau Thygesen, Søren T. Skou

**Affiliations:** 1 Centre for Health, Exercise and Sports Medicine, Department of Physiotherapy, The University of Melbourne, Melbourne, Australia; 2 Department of Physiotherapy and Occupational Therapy, The Research and Implementation Unit PROgrez, Slagelse, Denmark; 3 Department of Sports Science and Clinical Biomechanics, Research Unit for Musculoskeletal Function and Physiotherapy, University of Southern Denmark, Odense, Denmark; 4 Sydney Musculoskeletal Health, The Kolling Institute, School of Health Sciences, Faculty of Medicine and Health, University of Sydney, Sydney, Australia; 5 Department of Sports Science and Clinical Biomechanics, Research Unit for Exercise Epidemiology, Center of Research in Childhood Health, University of Southern Denmark, Odense, Denmark; 6 National Institute of Public Health, University of Southern Denmark, Odense, Denmark

**Keywords:** Exercise, Comorbidities, Sleep, Osteoarthritis, Quality of life, Physical function

## Abstract

**Purpose:**

This study investigated the associations between physical activity, sleep, and joint pain intensity with physical function and health-related quality of life (HrQoL) in people with multimorbidity.

**Methods:**

A pre-specified cross-sectional study using baseline data from participants with multimorbidity in the MOBILZE trial. Outcomes were physical function (6-minute walk test) and HrQoL (EQ-5D-5 L). Exposures were device-measured physical activity (min/week), joint pain intensity (0-100 visual analogue scale), device-measured sleep efficiency (%), and sleep quality (Modified Karolinska Sleep Questionnaire, 0–5 Likert scale). Separate and combined linear regression models were used to assess associations between exposures and outcomes.

**Results:**

We included data from 227 participants. Physical activity was positively associated with physical function (β = 0.56 m, 95% confidence intervals (CI) 0.39 to 0.73) and HrQoL (β = 0.00046, CI 0.000054 to 0.00087). Joint pain intensity was negatively associated with physical function (β = -0.90 m, CI -1.61 to -0.19), but not quality of life. Poorer sleep quality was negatively associated with HrQoL (β = -0.037, CI -0.060 to -0.013), but not physical function. Sleep efficiency was not associated with either outcome. In the combined model, physical activity was not associated with HrQoL, while joint pain was.

**Conclusions:**

Our exploratory findings among people with multimorbidity suggest that physical activity and joint pain are potentially clinically meaningful factors relating to physical function, while self-reported sleep quality may meaningfully relate to HrQoL. Clinicians may want to consider supporting patients with multimorbidity in managing their physical activity levels, joint pain, and sleep quality to improve health outcomes. Longitudinal studies and randomised controlled trials are needed to confirm these findings.

**Supplementary Information:**

The online version contains supplementary material available at 10.1007/s11136-025-04044-4.

## Background

Multimorbidity, defined as two or more long-term conditions in the same individual [1, 2], is common, affecting about one in three adults [3, 4]. Compared to individuals without multimorbidity, patients with comorbidities are more likely to functionally decline, develop depression, and experience poorer health-related quality of life [5–7]. Multimorbidity is also costly [2]. Those with four or more chronic conditions have almost 15 times the odds of experiencing an unplanned hospitalisation than those without multimorbidity [8]. Given these impacts and the projected increase in multimorbidity over the coming decades [9], there is a global need to better understand its personal and social burden.

Historically, healthcare for chronic conditions has centred on managing single conditions. This practice can fragment care for people with multimorbidity, increasing healthcare inefficiencies and treatment burdens [2, 10, 11]. Therefore, we need to improve health care for multimorbidity, but there are limitations and gaps in the evidence hampering our ability to do so. Studies of lifestyle interventions (e.g. exercise) often exclude people with multimorbidity [12–14]. A recent Cochrane review highlighted a lack of evidence for effective interventions, including lifestyle interventions, for people with multimorbidity [15].

There is also limited observational evidence about whether and to what extent lifestyle behaviours relate to health outcomes in people with multimorbidity. Physical activity and sleep quality are positively associated with health outcomes, such as physical function and health-related quality of life, in adults (45 years and older), including those with long-term conditions [16–18]. However, these relationships may differ among those with multimorbidity.

Joint pain may also negatively impact the health of individuals with multimorbidity. People with multimorbidity are more likely to have joint pain [19]. Individuals with multimorbidity and persistent pain may experience poorer health-related quality of life [20]. While hip and knee pain are two of the most common and burdensome types of joint pain [21; 22], there is a lack of evidence investigating how hip and knee pain intensity specifically relates to physical function and health-related quality of life in those with multimorbidity. Furthermore, given that people with joint pain are often less physically active and experience poorer sleep quality, [23; 24] it is important to understand if these factors are associated with health outcomes among people with multimorbidity and joint pain.

To develop effective interventions for individuals with multimorbidity, a better understanding of how physical activity, sleep and pain relate to their health is needed. Such research could inform treatment recommendations (i.e. help identify important treatment targets) and identify areas for future clinical research for multimorbidity. Therefore, we conducted this study to investigate the associations between physical activity, sleep, and joint pain severity with physical function and health-related quality of life in people with multimorbidity. Given that the relationship between physical activity and sleep with health outcomes may differ in those with joint pain compared to those without it, as a secondary aim, we investigated the associations between physical activity and sleep with physical function and health-related quality of life in those with multimorbidity and joint pain.

## Methods

### Study design

A cross-sectional observational study. This study was reported according to the STROBE checklist for observational studies (See eTable 1 in the supplement) [25]. The protocol for this paper was published on the Open Science Framework (https://osf.io/7tj36/). Ethics approval for this study was provided by the Regional Committee on Health Research Ethics for Region Zealand (SJ-857) on May 28th, 2020.

### Participants

This study uses baseline data from the MOBILIZE randomised controlled trial (RCT) for 227 participants with multimorbidity (i.e., data collected before participants either started the intervention or were assigned to usual care) [26]. Participant recruitment was guided by findings from a systematic review and Cochrane guidelines [26]. The recruitment began in November 2021 and was completed in the second quarter of 2023 (12-month follow-up completed mid-2024). Participants were recruited from general practitioners, psychiatric facilities, and hospital departments in the Region of Zealand, Denmark. All participants were recruited through direct consultations when visiting a recruitment site and presented with information about the study by a healthcare professional. Recruitment also occurred with advertisements via Facebook, poster advertisements, and handouts. Individuals expressing interest in the study, after viewing an advertisement, were contacted via phone to provide information about the study and were screened by the project researcher. These participants were assessed for exclusion criteria by a MOBILZE-associated health professional. Participants were encouraged to consider their enrolment in this trial for at least 24 h and discuss it with others (e.g., family) before consenting to it. All participants signed a written consent form for participation in this trial.

Eligible participants for the MOBILIZE RCT:

1. Had ≥ 2 of the following conditions: knee or hip osteoarthritis, chronic obstructive pulmonary disease, heart disease (heart failure or chronic heart disease), hypertension, type two diabetes mellitus, depression (having other comorbidities does not exclude a person from participating).
2. Were aged 18 years or above.
3. Able to walk three meters without assistance.
4. Had a score of ≥ 3on the Bayliss Disease Burden: Morbidity Assessment by Self-Report scale [27] for at least one of the conditions listed above and a score of ≥ 2 for at least one of the other listed conditions.
5. Were willing and able to participate in a 12-week supervised exercise therapy- and self-management program twice a week.

Participants were excluded from the MOBILIZE trial if they:

1. Participated in supervised systematic exercise for one of their diseases within the last 3 months.
2. Had an unstable health condition or at risk of serious adverse events as assessed by a medical specialist.
3. Were terminal patients and persons with a life expectancy of less than 12 months.
4. Were categorised as Class IV on the New York Heart Association (NYHA) Functional Classification scale [28].
5. Had psychosis disorders, post-traumatic stress disorder, obsessive compulsive disorder, attention deficit hyperactivity disorder, autism, anorexia nervosa/bulimia nervosa and/or persons with dependency disorders.
6. Had other reasons for exclusion (unable to understand Danish or cognitively unable to participate.)

### Outcomes

Participants included in the study were sent the baseline questionnaire and invited to complete in-person testing (e.g., physical performance testing). During this in-person testing, participants were also fitted with the accelerometers to measure physical activity and sleep levels (reported in the next section). On average, the in-person testing occurred about 18 days after participants completed baseline questionnaires. Our outcomes of interest were physical function and health-related quality of life. Physical function was measured with the 6-minute walk test (6MWT), a validated and reliable measure of walking function in people with chronic diseases [29, 30]. Health-related quality of life was measured with the EQ-5D-5 L, a validated measure in people with chronic diseases; it ranges from − 0.757 to 1, with higher scores indicating higher self-reported health-related quality of life [31].

### Exposures

The exposures were device-measured physical activity levels, device-measured sleep efficiency, self-reported sleep quality, and joint pain intensity. Physical activity was measured with two Axivity AX3 (Axivity, Newcastle UK) accelerometers placed on the front of the right thigh and the non-dominant wrist. Physical activity intensities are calculated from the thigh device using ActiGraph counts [32]. These data were used to calculate our independent variable of minutes spent in physical activity of at least light intensity using a threshold of 100 counts per minute over one week (original data were measured in seconds per week). Non-wear was identified in the acceleration using temperature and acceleration as described in the study by Rasmussen et al. 2020 [33]. Only two hours of non-wear is allowed for a day to be valid, and at least 14 h of recording for the first day is required for a day to be valid. Days with shorter awake time than 10 h were excluded. At least three valid weekdays and one weekend day are required to have a valid week. The estimated time spent in physical activity above light intensity is a weighted average using 5/7 for weekdays and 2/7 for weekend days. Sleep parameters were estimated validated algorithm HDCZA using the open-source R GGIR package and data from the wrist accelerometer (i.e. also using data from a seven-day period) [34]. Based on these data, sleep efficiency—actual sleep time divided by total time in bed multiplied by 100—was calculated. Self-reported sleep quality was measured with the Modified Karolinska Sleep questionnaire, with higher scores indicating worse sleep quality (measured on a 0–5 Likert scale) [35]. Finally, joint pain intensity of the most affected knee or hip (i.e. for those with knee and/or hip osteoarthritis) was measured using a 100-point visual analogue scale, with higher scores indicating greater pain intensity. The process of data collection is depicted in eFigure 1.

### Covariates

We generated Directed Acyclic Graphs (DAGs) with Dagitty (https://www.dagitty.net/dags.html#), guided by discussions within the project team, including researchers and clinician-researchers, to identify potential confounding variables informed by medical literature (see eFigure 2, eFigure 3, 2, eFigure 4, and eFigure 5 in the supplement) [2, 36–53]. A narrative summary is available in the supplement describing the selection of these variables (see eFile 1 in the supplement). The following covariates were selected: age, sex, body mass index, educational level, smoking habits, civil stats, and the number of comorbidities. Educational level was categorised into low (no finalised education, primary or secondary school) and higher education (vocational, short-term, middle-term, long-term education) (binary, low/higher).

### Data analysis

Participants’ characteristics were summarised using descriptive statistics and presented as means and standard deviations (SDs) or medians and interquartile range (IQR). Participant characteristics were tabulated across those with and without joint pain; we did this because relationships between the exposures and outcomes may differ between those with joint pain and those without it, as explored in a sensitivity analysis. All data analyses were performed with the software R (R Core Team, 2024). We used separate linear regression models to determine the relationships between physical activity, sleep efficiency, self-reported sleep quality, and joint pain intensity (exposures) with physical function and health-related quality of life (outcomes). We also performed a combined multivariable analysis for each outcome, physical function and health-related quality of life, which included three exposures: physical activity, sleep efficiency, and joint pain intensity. This model was performed to assess whether potential associations between these exposures and each outcome were influenced by the presence of other exposures. We did not include sleep quality in this pre-specified model, as we sought to better understand the relationships between a device-measure of sleep—an exposure less commonly studies than self-reported sleep—with health outcomes in multimorbidity. Associations were reported with 95% confidence intervals. The residuals of each linear model with diagnostic plots were used to check that the models did not violate relevant assumptions, such as normality. Sleep quality, which is an ordinal outcome, was treated as a continuous variable in the linear regression model. To assess the appropriateness of this assumption, we also visually inspected any observed associations between sleep quality and the outcomes using scatterplots to check if the relationships were broadly linear. Multicollinearity between independent variables was assessed for the multivariable models, with a threshold of less than *r* = 0.7 deemed acceptable [54].

In an exploratory, hypothesis generating analysis, to determine if the associations between physical activity and sleep efficiency with physical function and health-related quality of life differ among people with multimorbidity with joint pain compared to those without joint pain, we fit and qualitatively compared separate linear regression models for these participant groups. When a potentially nontrivial association was observed between an exposure and outcome, we calculated and plotted the E-value for these estimates (using the E-value calculator [55, 56]) to determine the minimum size of an association of an unmeasured confounder that would negate the observed association. While E-values need to be interpreted in the particular study context (e.g., how many potential confounding variables are included in the models and the potential size of unobserved confounders), in biomedical sciences, a variable affecting an exposure and outcome by 2 or 3 fold is likely not particularly common [56].

## Results

**Table 1:** Participant characteristics across those with and without joint pain, reported as mean (SD) unless otherwise stated

	Pain in hips and/or knees (*n* = 170)	No pain in hips and/or knees (*n* = 57)	Overall (*n* = 227)
Age (years)	70 (8.6)	70 (8.0)	70 (8.4)
Sex (%)^a^			
Female	81 (48)	17 (30)	98 (43)
Male	89 (52)	40 (70)	129 (57)
BMI (kg.m^2^; median [Q1, Q3])	31 (27, 34)	29 (26, 33)	30 (27, 34)
Numbers of comorbidities (median [Q1, Q3])	8 (6, 9)	6 (5, 8)	7 (5, 9)
Device measured physical activity levels of low intensity* or greater (minutes/week)	169 (61.2) (range 48 to 361)	160 (64.3) (range 49 to 337)	167 (62.0) (range 48 to 361)
Pain intensity (VAS: 0-100; median [Q1, Q3])	50.0 (31.0, 65.0) (range 9 to 95)	N/A	N/A
Device measured sleep efficiency^#^ (%)	84 (7.2) (range 41 to 96)	84 (8.2) (39 to 94)	84 (7.9) (range 39 to 96)
Sleep Quality^a,^^	2.00 (1.3, 3.0) (range 0 to 5)	2.00 (1.0, 2.8) (range 0.25 to 5)	2.00 (1.1, 2.9) (range 0 to 5)
Smoking (%)^a^			
Yes	19 (11)	5 (9)	24 (11)
No	151 (89)	52 (91)	203 (89)
Education (%)^a^			
No finalised education	11 (7)	1 (2)	12 (5)
Primary school	21 (12)	8 (14)	29 (13)
Secondary School	3 (2)	5 (9)	8 (4)
Vocational training	56 (33)	19 (33)	75 (33)
Short-term higher education (2–3 years)	22 (13)	9 (16)	31 (14)
Medium-term higher education (3–4 years)	44 (26)	12 (21)	56 (25)
Long-term higher education (> 4 years)	13 (8)	3 (5)	16 (7)
Civil status (%)^a^			
Single or never married	13 (8)	3 (5)	16 (7)
Married or cohabitating	119 (70)	36 (63)	155 (68)
Divorced	19 (11)	10 (18)	29 (13)
Widow	17 (10)	8 (14)	25 (11)
Separated	2 (1)	0 (0)	2 (1)

BMI: body mass index; VAS: Visual analogue scale

^a^Percentages may not equal 100 due to rounding

*calculated using a threshold of 100 counts per minute over one week

^#^sleep efficiency calculated as hours-asleep/time-in-bed*100

^measured with the Modified Karolinska Sleep questionnaire items for sleep quality, score from zero to five, with higher numbers indicating worse sleep quality

Data from 227 participants were included in this study. Participants are described in Table 1. Overall, most participants were male (57%), married or cohabitating (68%), had a median BMI of 30 kg/m^2^, and, on average, seven co-existing conditions or diseases. One hundred and seventy participants experienced hip and/or knee pain, with a median intensity of 50 out of 100 on a VAS. Participants with and without hip or knee pain were broadly similar in most characteristics. However, a larger proportion of those without hip or knee pain were males (70%) compared to those with hip or knee pain (52%). Characteristics were similar between participants with complete and missing data, except for civil and educational status; participants with missing data were often higher educated and divorced. 

No imputation was used for missing data for two key reasons. First, the amount of missing data was relatively low, ranging from < 1 to 7% (see eTable 2). Second, we suggest that most of the data were missing at random. This assertion is evidenced by the reasons the data was missing—mainly due to insufficient accelerometer wear time, for example, due to skin itching (see eTable 3). Furthermore, those for whom we did and did not have complete accelerometry data were broadly similar in most characteristics; potentially notable differences were that those with missing data, on average, had less pain, higher education, and were more likely to be divorced (see eTable 4).

### Findings of the separate linear regression models

Table 2 describes the findings for separate linear regression models, estimating the associations between physical activity levels and joint pain severity with physical function and health-related quality of life. Regression coefficients showed that physical activity levels were positively associated with physical function, with each additional minute of lower intensity or greater physical activity associated with a 0.56 m increase in the 6MWT (95% Confidence Intervals (CI) 0.39 to 0.73). Physical activity levels were also positively associated with health-related quality of life, with each additional minute of lower intensity or greater physical activity associated with a 0.00046 unit improvement on the EQ-5D-5 L (95% CI 0.000054 to 0.00087). Joint pain intensity was negatively associated with physical function, with each 1-unit of pain increase (on a 0-100 VAS) associated with a 0.90 m reduction in the 6MWT (95% CI -1.61 to -0.19). Joint pain intensity was not associated with health-related quality of life.

Table 3 describes the findings for the linear regression models for sleep efficiency and sleep quality with health outcomes. Sleep efficiency was not associated with either health outcome and sleep quality was not associated with physical function. Poorer sleep quality was negatively associated with health-related quality of life, with each 1 unit (on a 0–5 Likert scale) increase of poorer sleep quality associated with a 0.037 unit reduction in health-related quality of life (95% CI -0.060 to -0.013).

**Table 2:** Linear relationships between physical activity and joint pain severity with health outcomes

Outcome	Physical activity levels^a^					Joint pain intensity^b^				
	Observations (*n*)	Adj *R*^2^	Regression coefficient (β)	Regression coefficient (95% CI)	*p* value	Observations (*n*)	Adj *R*^2^	Regression coefficient (β)	Regression coefficient (95% CI)	*p* value
Physical function^c^	209	0.35*	0.56	0.39 to 0.73	< 0.001	166	0.28^#^	-0.90	-1.61 to -0.19	0.013
Health-related quality of life^2^	214	0.029^^^	0.00046	0.000054 to 0.00087	0.027	170	0.10^#^	-0.0013	-0.0030 to 0.00037	0.12

CI: confidence intervals

^a^Device measured minutes of low intensity or greater physical activity per week. Positive coefficients indicate increased physical activity is associated with increased physical function and health-related quality of life

^b^Measured on a visual analogue scale ranging from 0-100. Negative coefficients indicate worsening pain is associated with reduced physical and health-related quality of life

^c^Measured with the 6-minute walk test min in meters

^d^Measured with the EQ-5D-5 L questionnaire

*Adjusted for sex, educational level, smoking status, civil status, body mass index, and age

^^^Linear regression model adjusted for sex, educational level, smoking status, civil status, and age

^#^Linear regression model adjusted for sex, educational level, smoking status, civil status, body mass index, age, and comorbidities

**Table 3:** Linear relationships between sleep efficiency and sleep quality with health outcomes

	Sleep efficiency					Sleep Quality					
**Outcomes**	**Observations (n)**	**Adj R** ^**2^**^	**Regression coefficient (β)**	**Regression coefficient (95% CI)**	***p value***	**Observations (n)**	**Adj R** ^**2**^^	**Regression coefficient (β)**	**Regression coefficient (95% CI)**	***p value***	
Physical function^c^	207	0.22	1.12	-0.61 to 2.85	0.20	221	0.24	-9.74	-19.86 to 0.38		0.059
Health-related quality of life^d^	212	0.025	-0.00047	-0.0044 to 0.0034	0.83	226	0.056	-0.03 7	-0.060 to -0.013		0.0024

CI: confidence intervals

^a^Device measured sleep-efficiency is hours-asleep/time-in-bed*100. Positive coefficients indicate increased sleep efficiency is associated with increased physical function and health-related quality

^b^Self-reported sleep quality is measured with the Modified Karolinska Sleep Questionnaire. Negative coefficients indicate worse sleep quality is associated with reduced physical function and health-related quality of life

^c^Measured with the 6-minute walk test min in metres

^d^Measured with the EQ-5D-5 L questionnaire

^Linear regression model adjusted for sex, educational level, smoking status, civil status, body mass index, and age

### Findings of the combined linear regression models

Table 4 describes the findings of the combined linear regression models estimating the associations between physical activity, sleep efficiency, and joint pain intensity with physical function and quality of life. Physical activity levels were positively associated with physical function, with each additional minute of lower intensity or greater physical activity associated with a 0.44 m increase in the 6MWT (95% CI 0.24 to 0.65). Physical activity levels were not associated with health-related quality of life. Joint pain intensity was negatively associated with physical function, with each 1-unit of pain (on a 0-100 VAS) increase associated with a 1.00 m reduction in the 6MWT (95% CI -1.73 to -0.28). Joint pain intensity was negatively associated with health-related quality of life, with each 1 unit (on a 0-100 VAS) increase of pain intensity associated with a 0.0019 unit reduction in health-related quality of life (95% CI -0.0038 to -0.00011). Sleep efficiency was not associated with either outcome.

**Table 4:** Linear relationships between physical activity, sleep efficiency, and joint pain severity with health outcomes

			Physical activity^a^			Sleep efficiency^b^			Joint pain intensity^c^		
Outcomes	Observations (*n*)	Adj *R*^2^	Regression coefficient (β)	Regression coefficient (95% CI)	*p* value	Regression coefficient (β)	Regression coefficient (95% CI)	*p* value	Regression coefficient (β)	Regression coefficient (95% CI)	*p* value
Physical function^d^	155	0.31^*^	0.44	0.24 to 0.65	< 0.001	0.28	-1.51 to 2.07	0.79	-1.00	-1.73 to -0.28	0.0072
Health-related quality of life^e^	155	0.046^**#**^	0.00040	-0.000090 to 0.00090	0.11	-0.00092	-0.0055 to 0.0036	0.69	-0.0019	-0.0038 to -0.00011	0.038

CI: confidence intervals

*Linear regression model adjusted for sex, educational status, smoking status, civil status, and body mass index

^#^Linear regression model adjusted for sex, educational status, smoking status, civil status

^a^Device measured minutes of low intensity or greater physical activity per week. Positive coefficients indicate increased physical activity is associated with increased physical function and health-related quality of life

^b^Measured on a visual analogue scale ranging from 0-100. Negative coefficients indicate worsening pain is associated with reduced physical and health-related quality of life

^c^Device measured sleep-efficiency is hours-asleep/time-in-bed*100. Positive coefficients indicate increased sleep efficiency is associated with increased physical function and health-related quality

^d^Measured with the 6-minute walk test in meters

^e^Measured with the EQ-5D-5 L questionnaire

### Sensitivity analyses

We qualitatively compared the findings of the linear regression models for all participants with complete data (findings described above) with models including only people with joint pain. We only found a considerable difference in the association between physical activity levels and health-related quality of life, which was no longer significant when the models were performed with only people with hip and knee pain (see eTable 5 in the supplement for regression coefficients of these models). The E-values for the associations we observed in this study ranged from 1.05 to 1.62 (see eFigure 6, eFigure 7, eFigure 8, and eFigure 9 in the supplement for plots of these E-values). These suggest that confounding variables unobserved in our dataset, particularly socioeconomic factors (i.e. income and occupation), could potentially negate the observed associations.

## Discussion

Our findings provide exploratory evidence that physical activity, joint pain, and sleep quality may relate to physical function and health-related quality of life among those with multimorbidity. We observed that higher physical activity levels were positively associated with greater physical function and health-related quality of life. More severe hip or knee pain intensity was negatively associated with reduced physical function. Poorer self-reported sleep quality was negatively associated with health-related quality of life, while device-measured sleep efficiency was not associated with either health outcome. We observed similar associations when comparing separate models for physical activity, joint pain, and sleep with a model including all three exposures. These findings suggest that physical activity and joint pain may be, in part, independently associated with physical function. Lastly, when estimating associations with those with multimorbidity and joint pain, physical activity was no longer associated with health-related quality of life. Overall, associations ranged from potentially trivial to meaningful in size, with the exposures typically explaining more variance in physical function than health-related quality of life.

Our estimates suggest a potentially meaningful association between physical activity and physical function in individuals with multimorbidity. Based on the size of this association, higher physical activity of about 54 min weekly of any intensity may relate to a meaningful difference of 30.5 m in the 6MWT– determined using the minimal clinically important difference (MCID) for adults with pathologies (e.g. cardiorespiratory disease) [57]. These findings are consistent with previous literature investigating how physical activity impacts physical function in people with chronic illnesses. In a study of people with hip or knee osteoarthritis, most with multimorbidity, individuals with greater physical activity levels often self-reported higher physical function than those with lower physical activity levels [58]. Studies also show that adults over 60 who engage in more physical activity, including aerobic exercise, often function better, such as walking further and performing more activities of daily living [16; 18]. Our findings build on this previous research. Using device-measured physical activity rather than self-reported data– self-report measures are potentially limited in that they are prone to recall and social desirability bias [59, 60]– our findings may allow for a more explicit estimate of what volume of physical activity meaningfully relates to differences in physical function.

We found that joint pain severity, by about 34 points on a 100 VAS, may relate to a potentially meaningful difference in 6MWT performance (i.e. 30.5 m [57]). This builds on previous research showing that joint pain is negatively associated with self-reported physical function in people with multimorbidity [61, 62]. Our findings support the link between joint pain and physical function by demonstrating that joint pain may influence actual performance in a physical function task (i.e. increased changes in walking distance rather than self-report data). These findings, combined with those from previous studies, suggest an important area for future research. Studies with prospective designs and larger sample sizes could more rigorously inform our understanding of how physical activity and joint pain influence physical function in individuals with multimorbidity.

We found that self-reported sleep quality may meaningfully relate to health-related quality of life in people with multimorbidity, but device-measured sleep efficiency might not. Based on an MCID of 0.074 points for the EQ-5D in people with chronic diseases [63], our estimate suggests that experiencing poorer self-reported sleep quality, of two scores on a 0–5 Likert scale, is potentially meaningfully associated with a lower health-related quality of life. Previous literature supports our observation that device-measured and self-reported sleep quality may capture different patient information. Indeed, objectively measured and self-reported sleep often differ in what they estimate, such as sleep duration [64]; interestingly, these differences appear greater among those with health issues, such as poorer self-reported health, insomnia and depression [65]. These estimates also vary in how well they explain differences in health outcomes, such as quality of life and depression [17, 66]. Our findings provide further exploratory evidence supporting the need for future research to understand better how device-measured and self-reported sleep discreetly relate to health outcomes in people with multimorbidity. Such research could help clinicians choose measures of sleep that are most likely to relate to their patient’s health outcomes of interest.

While the association between joint pain and health-related quality of life was also significant in the combined multivariable model, with an estimate that is potentially clinically meaningful, the confidence intervals suggest considerable uncertainty around this estimate. Physical activity was also associated with health-related quality of life, but this association was potentially not meaningful. Our estimate suggested that about 160 min of additional physical activity would be required to achieve the MCID of 0.074 for the EQ-5D [63], which could be challenging to achieve for people with multimorbidity. Furthermore, among those with multimorbidity and joint pain, our sensitivity analysis suggested physical activity was unrelated to health-related quality of life in this subgroup, and all models in this study explained less than 3% of the variance in health-related quality of life. In contrast, associations were more consistent across models and sensitivity analysis for physical function. These models also explained considerably more variance in physical function (e.g. 35% in the adjusted physical activity model) than health-related quality of life. Taken together, our findings suggest that understanding which factors, such as lifestyle factors, relate to a person’s health-related quality of life might be more complex than for physical function. Given these findings and our knowledge that multimorbidity is negatively associated with health-related quality of life [5, 67], more research is needed to identify the factors contributing to health-related quality of life in this population. Such research could inform the design of interventions targeting health-related quality of life in individuals with multimorbidity.

Our findings provide exploratory evidence that could help inform clinicians about managing people with multimorbidity. Firstly, our findings suggest clinicians may want to consider supporting patients in increasing their physical activity. Even small increases in physical activity (e.g. an hour weekly) may relate to improved physical function. Given that most people with multimorbidity are not meeting recommended levels of physical activity [68], it is important that we find ways of supporting this patient group to become more physically active. The device used to capture the physical activity data for this study sought to capture all types of movement at lower intensities or greater. Therefore, these findings suggest that a range of physical activity and exercise types might be important for maintaining physical function. Clinicians may also want to adopt person-centred approaches when offering exercise therapy to patients with multimorbidity, such as tailoring physical activity and exercise to individual preferences.

The second clinical implication of our findings is that clinicians should consider helping patients with multimorbidity manage hip and knee pain if this aligns with what they want and need. Prior research suggests that those with hip and knee pain and increasing numbers of comorbidities often experience worse health status, including quality of life and walking function [69]. Addressing joint pain may enable patients to walk more [70], which could help them improve other health outcomes, such as self-rated health status and overall mortality [71, 72]. Of this patient group, those with more severe hip and knee pain, coexisting joint pain (e.g. back pain), and obesity may need extra clinical support from clinicians to help them improve their physical function [73]. However, RCTs are needed to rigorously evaluate if increasing physical activity and reducing joint pain improves physical function and health-related quality of life in people with multimorbidity.

Strengths of this research include using validated, device-measures of physical activity and sleep efficiency data. There are few studies of device-measured physical activity and sleep in adults with multimorbidity. Given the differences between device-measured and self-reported sleep, continued research in this area is important. Limitations of this research include its cross-sectional design and relatively small sample size. The calculated E-values suggest that most of the observed associations could be explained away by unobserved confounding variables, which have relatively small associations with both exposures and outcomes. These findings are potentially important given that we identified potential confounding variables (see eFigure 2, eFigure 3, eFigure 4, and eFigure 5) for which we had no data, such as participant occupation and income. Therefore, our findings should be interpreted with caution and treated as exploratory and hypothesis-generating, requiring investigation in larger longitudinal and experimental studies. Relating to sleep, the algorithm for calculating sleep efficiency was developed using participants without sleep problems [32]. It is possible that this algorithm may not perform optimally with data from individuals who often experience sleep problems. Similarly, an underrepresentation of people with multimorbidity in accelerometry validation studies may also mean that these devices are less accurate for measuring physical activity in this patient population [74]. Furthermore, participants may alter their physical activity behaviours because they know their physical activity is being monitored (i.e., wearing the accelerometer), which could bias the physical activity measurement [74]. Given the type of physical activity data used in this study, we could not determine if different types of intensity (e.g. moderate versus high) were more greatly associated with health outcomes. Lastly, we treated sleep quality (an exposure) as a continuous variable even though it is an ordinal variable. This decision was made to enable easier interpretation of the model, and the plotted relationship appeared approximately linear. However, we acknowledge that by doing so, the model assumes a linear relationship with the outcome and thus may not fully capture the true nature of the association.

## Conclusions

Our exploratory findings among people with multimorbidity suggest that physical activity and joint pain are potentially important factors relating to physical function, while self-reported sleep quality may meaningfully relate to health-related quality of life. Estimates of these associations suggest that plausible differences in physical activity, joint pain, and sleep quality could relate to important differences in health outcomes based on published MCIDs. Clinicians may want to consider supporting patients with multimorbidity to manage their physical activity levels, joint pain, and sleep quality to improve health outcomes. Longitudinal studies and RCTs are needed to confirm these findings and evaluate the effects of interventions targeting these factors.

## Supplementary Information

Below is the link to the electronic supplementary material.

Supplementary Material 1

## Data Availability

Data available on reasonable request.
