# Supplementary material for: Are physical activity, sleep, and joint pain associated with physical function and quality of life in individuals with multimorbidity? A cross-sectional analysis of the MOBILIZE trial
Source: Qual Life Res. 2025 Aug 9;34(12):3575–86. doi: 10.1007/s11136-025-04044-4 (PMC12689766; doi:10.1007/s11136-025-04044-4)
Supplement: Supplementary file 1 — Supplementary Material 1 [file 11136_2025_4044_MOESM1_ESM.docx]

eTable 1 STROBE checklist for observation study

STROBE Statement—Checklist of items that should be included in reports of ***cross-sectional studies***

|  | Item No | Recommendation | Page No |
| --- | --- | --- | --- |
| **Title and abstract** | 1 | (*a*) Indicate the study’s design with a commonly used term in the title or the abstract | 1,2 |
|  |  | (*b*) Provide in the abstract an informative and balanced summary of what was done and what was found | 1,2 |
| Introduction | | | |
| Background/rationale | 2 | Explain the scientific background and rationale for the investigation being reported | 4-5 |
| Objectives | 3 | State specific objectives, including any prespecified hypotheses | 5 |
| Methods | | | |
| Study design | 4 | Present key elements of study design early in the paper | 5 |
| Setting | 5 | Describe the setting, locations, and relevant dates, including periods of recruitment, exposure, follow-up, and data collection | 5 |
| Participants | 6 | (*a*) Give the eligibility criteria, and the sources and methods of selection of participants | 6-7 |
| Variables | 7 | Clearly define all outcomes, exposures, predictors, potential confounders, and effect modifiers. Give diagnostic criteria, if applicable | 7-8 |
| Data sources/ measurement | 8* | For each variable of interest, give sources of data and details of methods of assessment (measurement). Describe comparability of assessment methods if there is more than one group | 7-8 |
| Bias | 9 | Describe any efforts to address potential sources of bias | 9-10 |
| Study size | 10 | Explain how the study size was arrived at | N/A |
| Quantitative variables | 11 | Explain how quantitative variables were handled in the analyses. If applicable, describe which groupings were chosen and why | 9-10 |
| Statistical methods | 12 | (*a*) Describe all statistical methods, including those used to control for confounding | 9-10 |
|  |  | (*b*) Describe any methods used to examine subgroups and interactions | 9-10 |
|  |  | (*c*) Explain how missing data were addressed | 9-10 |
|  |  | (*d*) If applicable, describe analytical methods taking account of sampling strategy | 9-10 |
|  |  | (*e*) Describe any sensitivity analyses | 9-10 |
| Results | | | |
| Participants | 13* | (a) Report numbers of individuals at each stage of study—eg numbers potentially eligible, examined for eligibility, confirmed eligible, included in the study, completing follow-up, and analysed | 11 |
|  |  | (b) Give reasons for non-participation at each stage | N/A |
|  |  | (c) Consider use of a flow diagram | N/A |
| Descriptive data | 14* | (a) Give characteristics of study participants (eg demographic, clinical, social) and information on exposures and potential confounders | Table 1, 11 |
|  |  | (b) Indicate number of participants with missing data for each variable of interest | Table 2, 3, 4 |
| Outcome data | 15* | Report numbers of outcome events or summary measures | N/A |
| Main results | 16 | (*a*) Give unadjusted estimates and, if applicable, confounder-adjusted estimates and their precision (eg, 95% confidence interval). Make clear which confounders were adjusted for and why they were included | 10-12, Table, 2, 3, 4 |
|  |  | (*b*) Report category boundaries when continuous variables were categorized | 9, Table, 2, 3, 4 |
|  |  | (*c*) If relevant, consider translating estimates of relative risk into absolute risk for a meaningful time period | N/A |
| Other analyses | 17 | Report other analyses done—eg analyses of subgroups and interactions, and sensitivity analyses | 12, 13, Supplementary |
| Discussion | | | |
| Key results | 18 | Summarise key results with reference to study objectives | 13 |
| Limitations | 19 | Discuss limitations of the study, taking into account sources of potential bias or imprecision. Discuss both direction and magnitude of any potential bias | 13-17 |
| Interpretation | 20 | Give a cautious overall interpretation of results considering objectives, limitations, multiplicity of analyses, results from similar studies, and other relevant evidence | 13-17 |
| Generalisability | 21 | Discuss the generalisability (external validity) of the study results | 13-17 |
| Other information | | | |
| Funding | 22 | Give the source of funding and the role of the funders for the present study and, if applicable, for the original study on which the present article is based | 17 |

*Give information separately for exposed and unexposed groups.

**Note:** An Explanation and Elaboration article discusses each checklist item and gives methodological background and published examples of transparent reporting. The STROBE checklist is best used in conjunction with this article (freely available on the Web sites of PLoS Medicine at http://www.plosmedicine.org/, Annals of Internal Medicine at http://www.annals.org/, and Epidemiology at http://www.epidem.com/). Information on the STROBE Initiative is available at www.strobe-statement.org

^
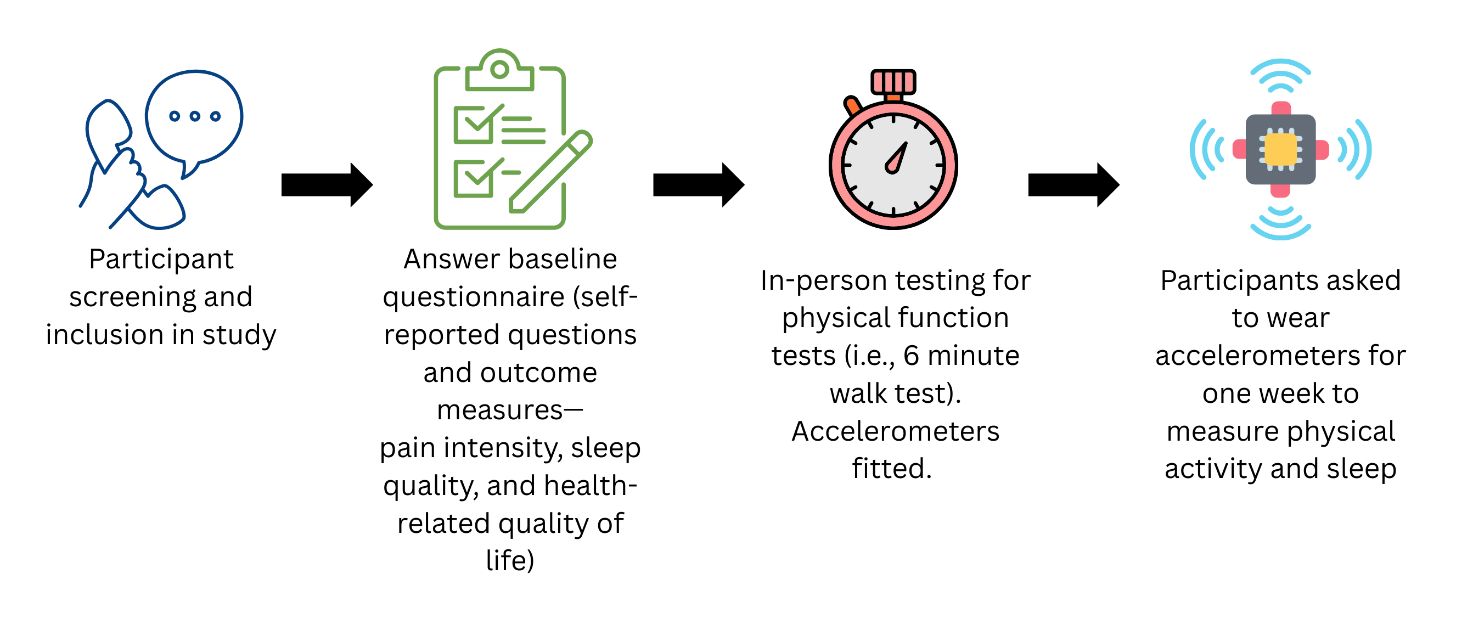
eFigure 1. Baseline data collection for the MOBILIZE randomised controlled trial^


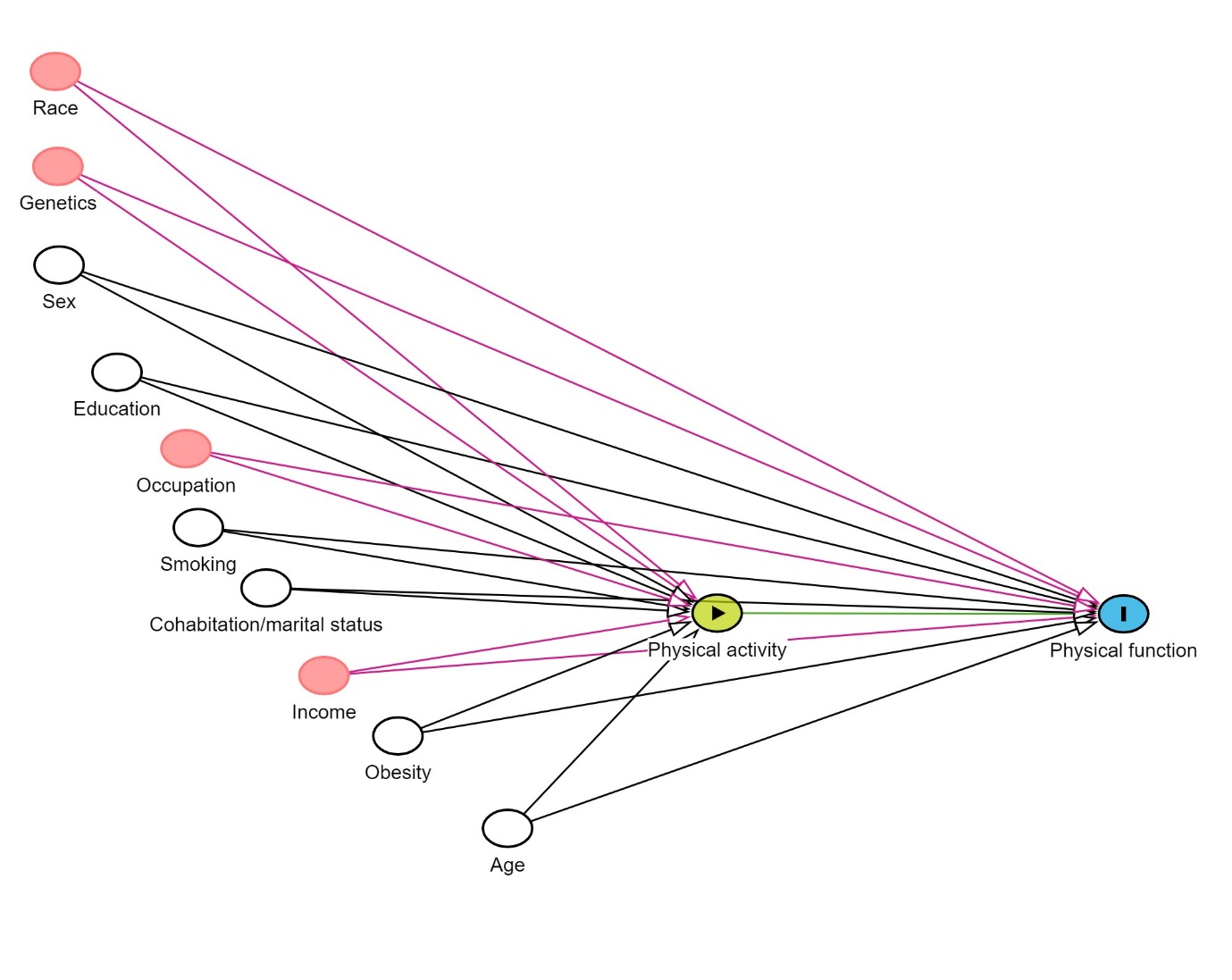


eFigure 2. Direct acyclic graph (DAG) showing the relationships between physical activity and physical function. The green arrow is the causal pathway. White circles are adjusted confounding factors. Red circles are potential confounding variables unadjusted in the linear regression models, with corresponding red arrows indicating potential biasing pathways. The DAG is temporally represented from left to right.


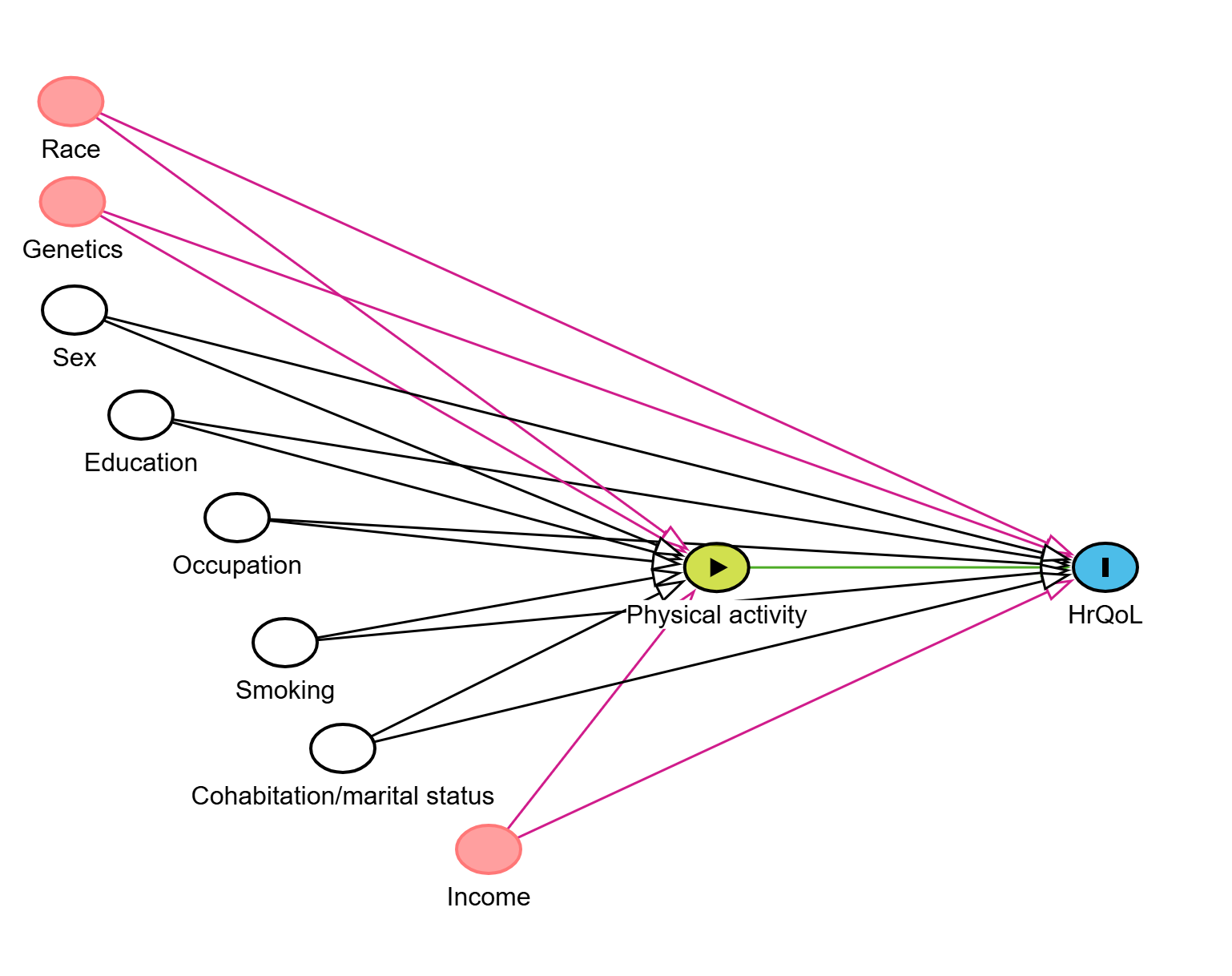


eFigure 3. Direct acyclic graph showing the relationships between physical activity and health-related quality of life (HrQoL). The green arrow is the causal pathway. White circles are adjusted confounding factors. Red circles are potential confounding variables unadjusted in the linear regression models, with corresponding red arrows indicating potential biasing pathways. The DAG is temporally represented from left to right.


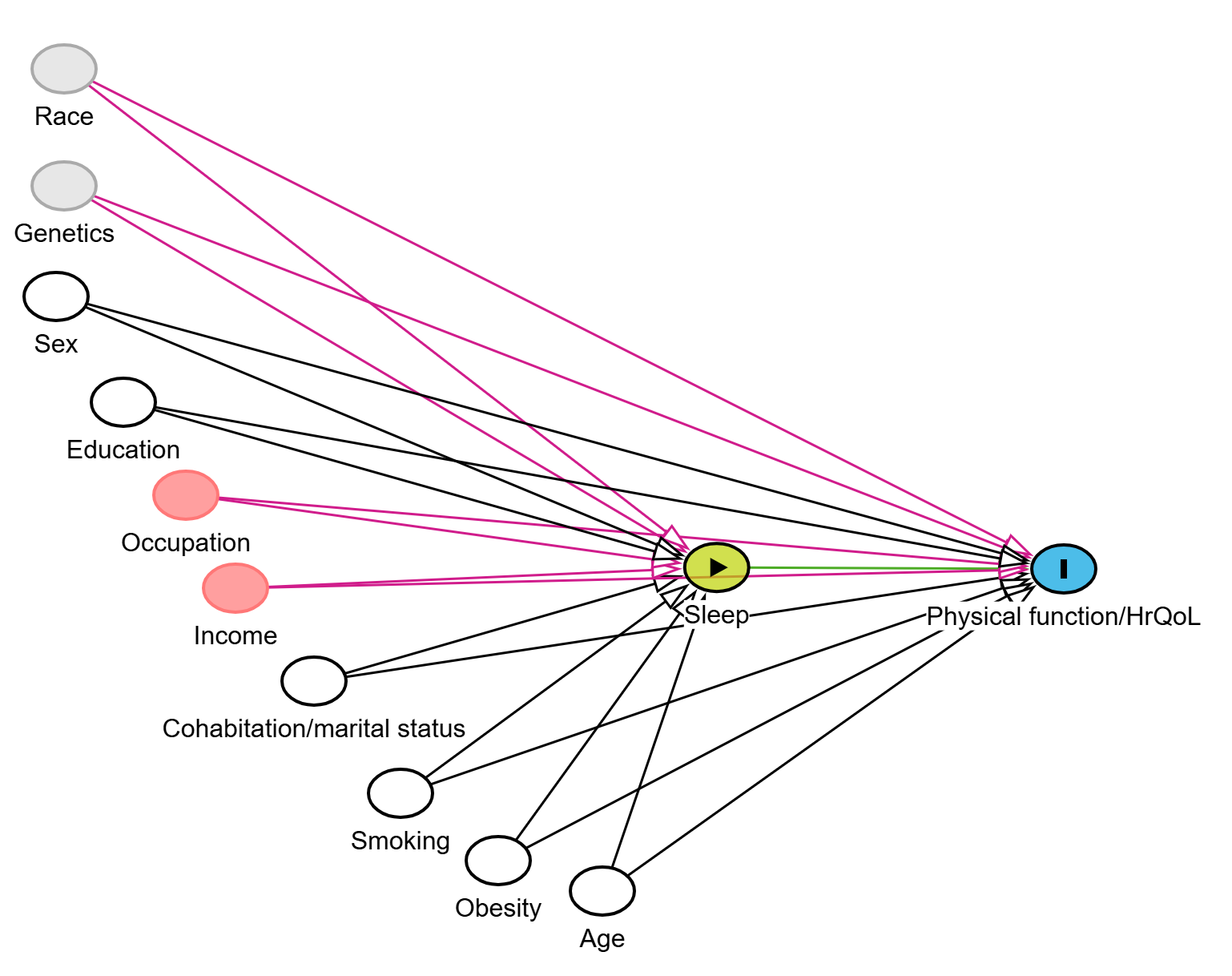


eFigure 4. Direct acyclic graph showing the relationships between sleep and physical function and health-related quality (HrQoL). The green arrow is the causal pathway. White circles are adjusted confounding factors. Red circles are potential confounding variables unadjusted in the linear regression models, with corresponding red arrows indicating potential biasing pathways. The DAG is temporally represented from left to right.


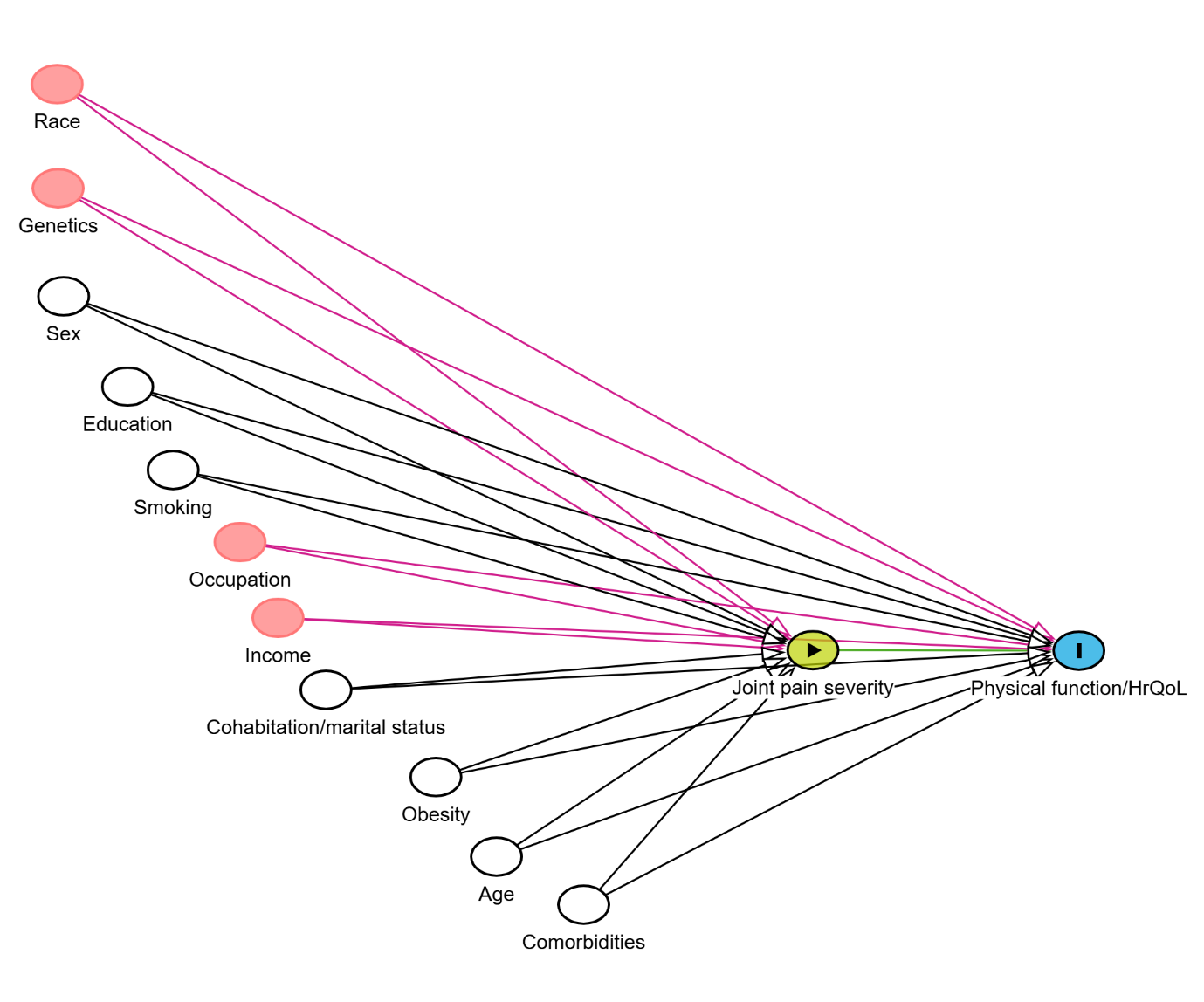


eFigure 5. Direct acyclic graph showing the relationships between joint pain severity and physical function and health related quality of life (HrQoL). The green arrow is the causal pathway. White circles are adjusted confounding factors. Red circles are potential confounding variables unadjusted in the linear regression models, with corresponding red arrows indicating potential biasing pathways. The DAG is temporally represented from left to right.

eFile 1. Selection of potentially confounding variables for the models

We selected covariates based on their potential to be confounding variables. These variables were selected based on expert knowledge within our research team and a review of medical literature. Below is a summary of the medical literature used to inform this selection. (Note: References for the following citations are included in the References section of the main text.)

For the model of physical activity and physical function, we included the covariates age, sex, education, smoking status, and marital status as potentially confounding variables for which we had data. Age is associated with reduced physical activity [38]. A systematic review (n=27 studies) and an umbrella review of systematic reviews (n=19 reviews) found that being male, not smoking, being more highly educated, and having social support are associated with increased physical activity levels.[38, 40] Socioeconomic factors, including educational status, were associated with self-reported physical function in a United States cohort of adults with knee osteoarthritis (n=782) [34]. For the model of physical activity and health-related quality of life, we included the covariates sex, educational level, smoking status, civil status, and age. The above-summarised evidence relating to physical activity is also relevant to this model. Relating to health-related quality of life, lower socioeconomic status (e.g., lower educational level) was negatively associated with health-related quality of life in adults in Germany (n=11,177), with about 1 in 4 of these adults reporting a chronic condition [43]. A survey of the French population (n=7525 men and 8486 women) found that smoking more than five cigarettes a day was negatively associated with health-related quality of life [42].

For the models including the exposures sleep efficiency or sleep quality and the outcomes physical function or health-related quality of life, we included the covariates sex, educational level, smoking status, civil status, body mass index, and age. Evidence for variables associated with self-reported physical function is summarised previously. For sleep quality, age was associated with being classified as a poorer sleep in a sample of older adults in rehabilitation (n=163) [35]. In a population-based case-control study in Germany (currently smoking: n=1071; never smokers: n=1243 ), smoking was negatively associated with self-reported sleep quality [37]. A systematic review and meta-analysis (including 19 studies) found that social status related to a range of device-measured sleep variables [44]. Notably, high educational status was positively associated with improved sleep efficiency [44]. Lower socioeconomic factors, including lower educational attainment, were also negatively related to various self-reported measures of sleep quality in adults [44]. A review of reviews with meta-analysis (n=12) found that obesity was negatively associated with obesity-specific and general measures of health-related quality of life in adults [45].

For the models of joint pain and physical function and joint pain and health-related quality of life, we included the covariates sex, educational level, smoking status, civil status, body mass index, number of comorbidities, and age. Evidence for an association between some of these variables and physical function and health-related quality of life is summarised previously. A number of comorbidities and body mass index are negatively associated with physical function in people with osteoarthritis and with the development of joint pain (body mass index: systematic review of 14 studies; comorbidities: cohort matched-controlled study of 221 807 incident OA cases and 221 807 matched control) [46-47]. In a cohort of people with knee osteoarthritis undergoing total knee joint replacement surgery, higher educational attainment and socioeconomic status (indicated using area-level socioeconomic status) were positively associated with lower pain levels [49]. In cohort studies (ranging from 398 to 2925 participants with health conditions), the number of comorbidities is shown to be negatively associated with health-related quality of life in adults with various conditions, including osteoarthritis, rheumatoid arthritis, and hypertension [48, 50-51].

eTable 2. Missing data for outcomes and exposures (n=227 participants)

| **Outcome/exposure** | **Number missing in whole data set (%)** |
| --- | --- |
| Walking function | 5 (2%) |
| Health-related quality of life | 0 (0%) |
| Physical activity levels | 13 (6%) |
| Sleep efficiency | 15 (7%) |
| Sleep Quality | 1 (<1%) |
| Joint pain | 2 (1%)* |
| *missing data is the proportion of 170 participants whom had hip/knee pain | |

eTable 3. Reasons for missing device-measured sleep and physical activity data

| Participant* | Thigh accelerometer (physical activity and sleep data) | Wrist accelerometer (sleep data) | Reasons for insufficient wear time |
| --- | --- | --- | --- |
| Person-1 | AX3 lost in the mail | AX3 lost in the mail |  |
| Person-2 |  | Not activated correct by a mistake |  |
| Person-3 | Don’t want to wear AX3 | Don’t want to wear AX3 |  |
| Person-4 | Withdrawn consent | Withdrawn consent |  |
| Person-5 | Wear time is too short according to the protocol | Wear time is too short according to the protocol | *Removed due to itching* |
| Person-6 | Error in data download | Error in data download |  |
| Person-7 | AX3 lost in the mail | AX3 lost in the mail |  |
| Person-8 | Wear time is too short according to the protocol | Wear time is too short according to the protocol | *Didn’t want to participate after all* |
| Person-9 | AX3 lost in the mail | AX3 lost in the mail |  |
| Person-10 |  | Not activated correct by a mistake |  |
| Person-11 |  | Wear time is too short according to the protocol | *Removed due to itching* |
| Person-12 |  | Wear time is too short according to the protocol | *Removed due to skin irritation* |
| Person-13 | Wear time is too short according to the protocol |  | *Fell off and was not put back on* |
| Person-14 | Wear time is too short according to the protocol | Wear time is too short according to the protocol | *Removed due to itching* |
| Person-15 | Wear time is too short according to the protocol |  | *Not specified* |
| Person-16 | Wear time is too short according to the protocol | Wear time is too short according to the protocol | *Didn’t want to participate after all* |
| Person-17 | Wear time is too short according to the protocol |  | *Not specified* |
| Person-18 | Wear time is too short according to the protocol | Wear time is too short according to the protocol | *Removed due to itching* |
| Person-19 | Wear time is too short according to the protocol | Wear time is too short according to the protocol | *Removed due to eczema* |
| Person-20 | Wear time is too short according to the protocol |  | *Not specified* |
| *Participants' actual study IDs were replaced with arbitrary IDs to adhere to the General Data Protection Regulation. | | | |

eTable 4. Participant data across participants with and without missing data

|  | **Participants without missing physical activity data (n=214)** | **Participants with missing physical activity data (n=13)** | **Participants without missing sleep efficiency data (n=212)** | **Participants with missing sleep efficiency data (n=15)** |
| --- | --- | --- | --- | --- |
| Age (years) | 70 (8) | 64(8) | 70 (8) | 65 (8) |
| Sex (%)^a^ |  |  |  |  |
| Female | 93 (44) | 5 (39) | 92 (43) | 6 (40) |
| Male | 121 (57) | 8 (61) | 120 (57) | 9 (60) |
| BMI (kg.m2; median [Q1, Q3]) | 30 (27, 34) | 32 (28, 34) | 31 (27, 34) | 29.5 (27, 33) |
| Numbers of comorbidities (median [Q1, Q3]) | 7 (5, 9) | 7 (6, 9) | 7 (5, 9) | 7 (6, 8) |
| Pain intensity (VAS: 0-100; median [Q1, Q3]) | 50 (37, 65) | 31 (29, 57) | 50 (39, 65) | 31.0 (20, 60) |
| Smoking (%)^a^ |  |  |  |  |
| Yes | 23 (10) | 1 (8) | 23 (11) | 1 (7) |
| No | 191 (89) | 12 (92) | 189 (89) | 14 (93) |
| Education (%)^a^ |  |  |  |  |
| No finalised education | 12 (6) | 0 (0) | 11 (5) | 1 (7) |
| Primary school | 27 (13) | 2 (15) | 28 (13) | 1 (7) |
| Secondary School | 7 (3) | 1 (8) | 7 (3) | 1 (7) |
| Vocational training | 71 (33) | 4 (30) | 71 (33) | 4 (27) |
| Short-term higher education (2-3 years) | 30 (14) | 1 (8) | 30 (14) | 1 (7) |
| Medium-term higher education (3-4 years) | 53 (25) | 3 (23) | 53 (25) | 3 (20) |
| Long-term higher education (> 4 years) | 14 (7) | 2 (15) | 12 (6) | 4 (26) |
| Civil status (%)^a^ |  |  |  |  |
| Single or never married | 15 (7) | 1 (8) | 16 (8) | 0 (0) |
| Married or cohabitating | 148 (69) | 7 (54) | 136 (69) | 9 (60) |
| Divorced | 24 (11) | 5 (39) | 23 (11) | 6 (40) |
| Widow | 25 (12) | 0 (0%) | 25 (12) | 0 (0%) |
| Separated | 2 (1) | 0 (0%) | 2 (1) | 0 (0%) |

BMI: body mass index; Visual analogue scale

^a^Percentages may not equal 100 due to rounding

*calculated using a threshold of 100 counts per minute over one week

eTable 5. Linear relationships between physical activity and sleep efficiency with health outcomes in those with multimorbidity and joint pain

|  | **Physical activity levels** | | | | | **Sleep efficiency** | | | | |
| --- | --- | --- | --- | --- | --- | --- | --- | --- | --- | --- |
|  | **Observations (n)** | **Slope (β)** | **Slope p value** | **Adj R^2^** | **Regression coefficient (95% CI)** | **Observations (n)** | **Slope** | **Slope p value** | **Adj R^2^** | **Regression coefficient (95% CI)** |
| **Physical function^1^** | 156 | 0.51 | 0.00028 | 0.30* | 0.31 to 0.71 | 153 | 1.04 | 0.20 | 0.20* | -0.86  to 2.95 |
| **Health-related quality of life^2^** | 160 | 0.00046 | 0.028 | 0.0029^^^ | -0.000023 to 0.00094 | 157 | -0.00031 | 0.85 | 0.036^^^ | -0.0047 to 0.0041 |
| ^a^Device measured minutes of low intensity or greater physical activity per week. Positive coefficients indicate increased physical activity is associated with increased physical function and health-related quality of life.  ^b^Device measured sleep-efficiency is hours-asleep/time-in-bed*100. Positive coefficients indicate increased sleep efficiency is associated with increased physical function and health-related quality.  ^c^Measured with the 6-minute walk test min  ^d^Measured with the EQ-5D-5L questionnaire  *adjusted for sex, education, smoking status, civil status, body mass index, and age  ^^^adjusted for sex, education, smoking status, civil status, and age | | | | | | | | | | |


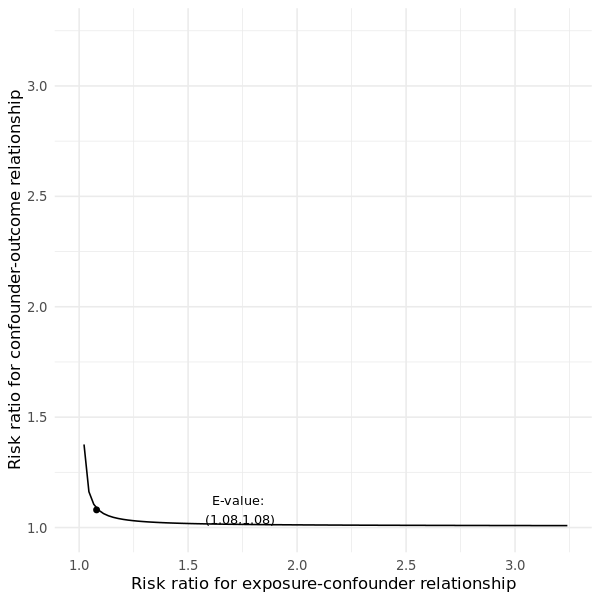


eFigure 6. E-values plot for association between physical activity and physical function


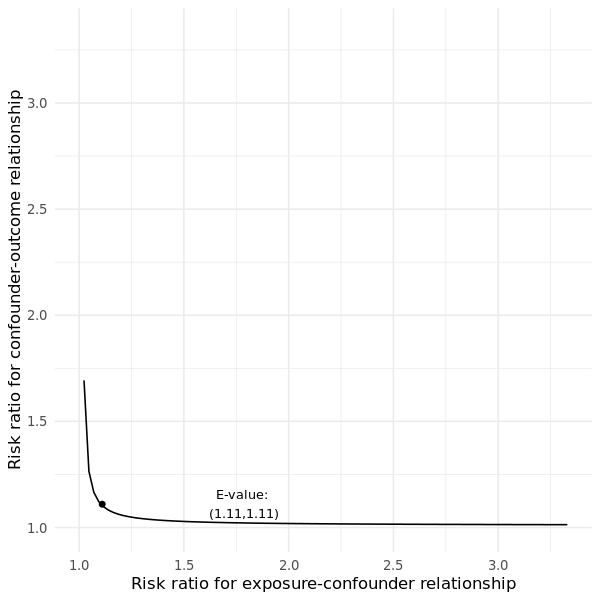


eFigure 7. E-values plot for association between joint pain and physical function


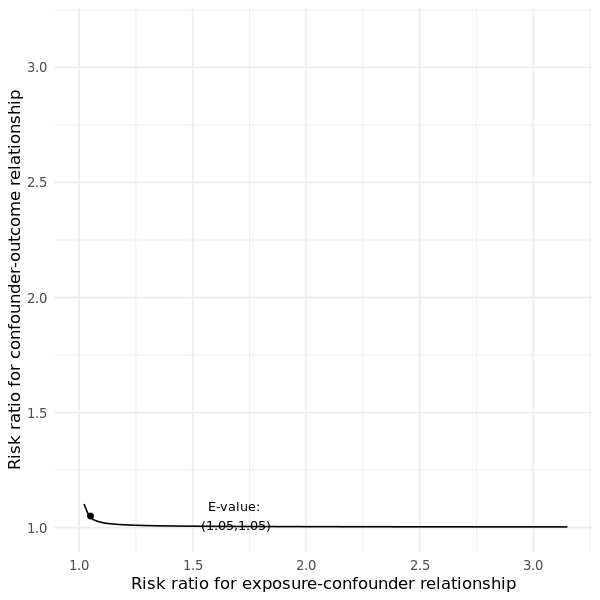


eFigure 8. E-values plot for association between physical activity and health-related quality of life


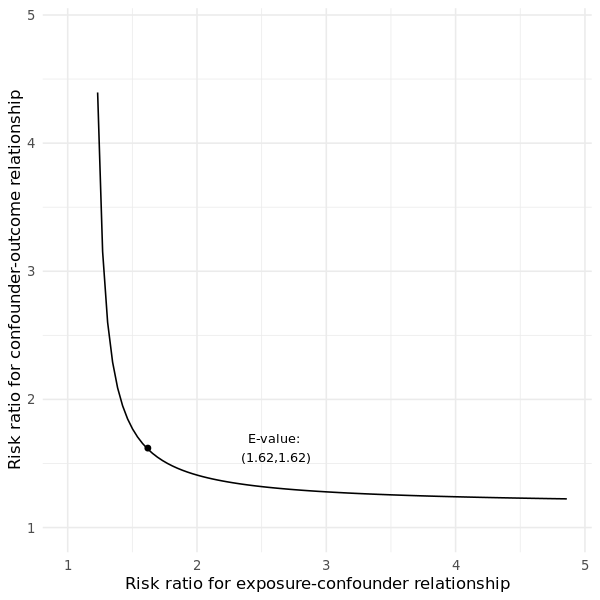


eFigure 9. E-values plot for association between self-reported sleep quality and health-related quality of life
